# Extracellular vesicles: what secrets do they hold inside?

**DOI:** 10.1038/s41419-019-1643-9

**Published:** 2019-05-28

**Authors:** Ishai Luz, Tomer Cooks

**Affiliations:** Faculty of Health Sciences, The Shraga Segal Department of Microbiology, Immunology and Genetics, Ben-Gurion University of the Negev, Beer-Sheva, Israel

**Keywords:** Cancer microenvironment, Extracellular signalling molecules

Recent years have seen the rise of unique cell-to-cell communication mechanisms in the form of extracellular vesicles (EVs). Among various biomolecules shipped between cells, different RNA species have been reported as key messengers. Importantly, although many of the extracellular RNA (exRNA) studies focus on microRNAs (miRNAs), EVs carry various types of RNA species, miRNA being just a small part of this repertoire^1,2^. Notably, various recent studies described the presence of exRNA in different biofluids. As biofluids are enriched with RNAses, the exRNA must be efficiently protected. Several studies suggested that, among several RNA protection mechanisms, the RNA could also be encapsulated within EVs^3–5^. Cancer cells use EVs as an efficient way of interacting with the tumor microenvironment, as well as with distant tissues. EV-mediated communication have been shown to promote a plethora of cancer-promoting traits, including immunosupression, invasion, and the nourishing of the metastatic niche^6–8^. On top of that, as vesicle-encased proteins and RNAs are of tumor-specific origin, they have been suggested as potential biomarkers and therapeutic targets.

**Fig. 1:**
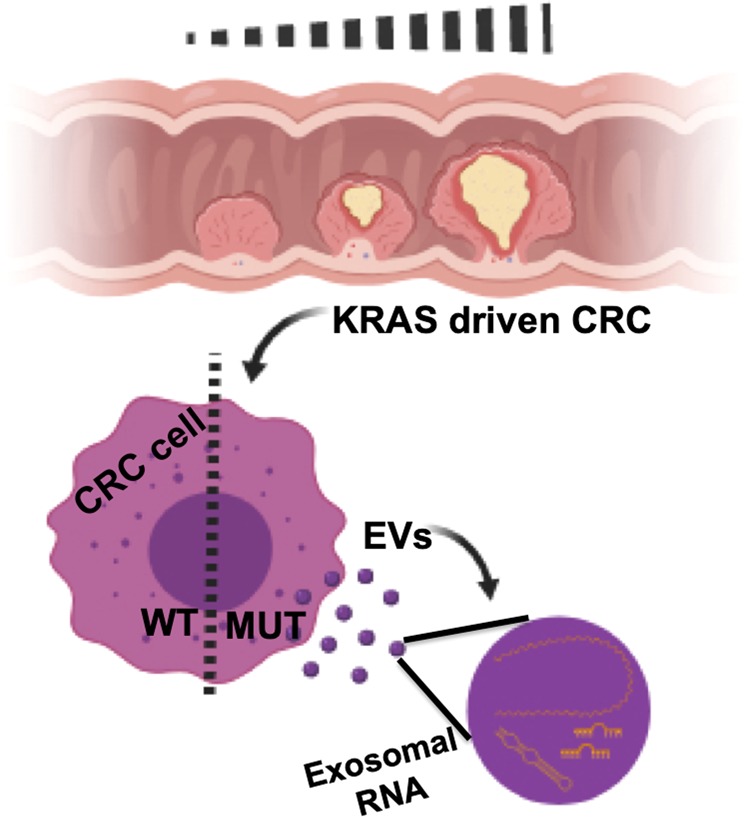
The presented study suggests a model for mutated-KRAS-driven colorectal cancer (CRC), based on specific signatures of RNA species sorted into exosomes (EVs). Besides the typical microRNAs, the authors focused on long coding and non-coding species

A new study by Hinger et al.^9^ addressed the intriguing question of specific sorting mechanisms of different RNA species into EVs, as a crucial step in understanding the downstream RNA-dependent oncogenic signaling and its biological and pathological role^9^. The study involved three derivatives of a colorectal cancer (CRC) cell line (DLD-1), differing by their KRAS status. EVs from these cells were previously compared and found to have distinct mutant KRAS-dependent EV cargo. The mutant-derived EVs were shown to be taken up by wild-type (WT) cells, consequently driving tumorigenesis^10,11^. In this present study, the researchers aimed to investigate the long-RNA molecules (over 200 nt) encased in the EVs of the three cell derivatives. They have previously determined that the ~40–130 nm EVs contain distinct proteomic and transcriptomic profiles, compared with both their parental cells and with EVs from WT-KRAS cells. In this current study, the authors used RNA-sequencing to analyze the long-RNA content in the same EVs (Fig. 1).

This mapping revealed that long-RNA sequences extracted from EVs poorly matched the human genome compared with the cellular RNA. These multiple mismatches were explained by the enrichment of modified RNAs in EVs. Yet, when the RNA profile was aligned specifically with annotated genes, a selective sorting mechanism into EVs was observed. Nevertheless, most of the RNA in EVs corresponded with unannotated regions. Interestingly, unlike the cellular content, EVs were enriched with non-coding transcripts of pseudogenes such as KRASP1, known to function as a Ras proto-oncogene^12^.

Long RNAs from different EVs were also found to differ by KRAS status, whereas the long RNAs in the cells themselves were not as diverse. The highest diversity in long-RNA populations was found in both the EVs and the cells with a single mutant allele of KRAS (DKO-1 cells). Unlike miRNAs that were previously shown to be KRAS status dependent, here most of the upregulated long RNAs were similar in all three cell lines. The authors used quantitative PCR to further investigate whether the mRNA profile found in the EVs represents full-length sequences. Several mRNAs known to relate to oncogenic genes, such as *Rab13*, were found to be upregulated and although most mRNA molecules were suggested to be fragmented, Rab13 was packaged and shipped at full length. Furthermore, transfer of *Rab13* between cells was noted to be unidirectional: *trans*-well experiments determined that *Rab13* mRNA transfers from the KRAS mutant cells to WT cells but not vice versa.

The last part of the study focused on the EV-mediated transfer of long non-coding RNA (lncRNA). Despite previous reports presenting lncRNA in EVs, the functional role is not yet elucidated. The research group used a CRISPR display system targeting lncRNAs to specific genomic sites accompanied by a reporting luciferase marker. In yet another series of *trans*-well experiments, the KRAS-mutated cells delivered lncRNA molecules to their recipient WT counterparts. Importantly, this effect was KRAS dependent, as such phenomenon was not observed with donor WT-KRAS cells.

The findings summarized in Hinger et al.^9^ address several key questions in the field of EVs in general, and most specifically in oncogenic mechanisms promoted by such cell-to-cell communication. The authors focused on a major genetic alteration frequently found in CRC. Mutations in KRAS play a central role in driving CRC tumorigenesis and characterizing the involvement of specific RNA cargo of EVs may shed light on such a malignant process. Similar to previous reports, this study suggests specific RNA enrichment in EVs that is selectively sorted. Furthermore, long coding and non-coding RNAs, including pseudogenes transcripts that their regulatory functions are not completely clear, are transferred between cells via EVs and this effect might be governed by mutant KRAS. The simplicity of the in vitro approaches used in this study allows us to decipher and study the RNA composition of each cell type. Taking these conclusions and studying them in a more complex setting, either in the tissue or organism level, might pose a much bigger challenge.

Nevertheless, these assays allow us to focus on the content of EVs, their specific RNA transcripts, and their ability to be exported and taken up by recipient cells. The fact that many lncRNAs are to be shipped via EVs indicates that these molecules hold significant facilitating roles yet to be acknowledged. This scientific effort is another stride in this direction.
